# A Conserved Mammalian Protein Interaction Network

**DOI:** 10.1371/journal.pone.0052581

**Published:** 2013-01-02

**Authors:** Åsa Pérez-Bercoff, Corey M. Hudson, Gavin C. Conant

**Affiliations:** 1 Smurfit Institute of Genetics, University of Dublin, Trinity College, Dublin, Ireland; 2 Informatics Institute, University of Missouri, Columbia, Missouri, United States of America; 3 Division of Animal Sciences, University of Missouri, Columbia, Missouri, United States of America; King Abdullah University of Science and Technology, Saudi Arabia

## Abstract

Physical interactions between proteins mediate a variety of biological functions, including signal transduction, physical structuring of the cell and regulation. While extensive catalogs of such interactions are known from model organisms, their evolutionary histories are difficult to study given the lack of interaction data from phylogenetic outgroups. Using phylogenomic approaches, we infer a upper bound on the time of origin for a large set of human protein-protein interactions, showing that most such interactions appear relatively ancient, dating no later than the radiation of placental mammals. By analyzing paired alignments of orthologous and putatively interacting protein-coding genes from eight mammals, we find evidence for weak but significant co-evolution, as measured by relative selective constraint, between pairs of genes with interacting proteins. However, we find no strong evidence for shared instances of directional selection within an interacting pair. Finally, we use a network approach to show that the distribution of selective constraint across the protein interaction network is non-random, with a clear tendency for interacting proteins to share similar selective constraints. Collectively, the results suggest that, on the whole, protein interactions in mammals are under selective constraint, presumably due to their functional roles.

## Introduction

Modeling genetic complexity in a network framework allows researchers to study the evolution of structures larger than a single gene [1]. While such efforts are confounded by the fact that the network is usually only known for a single taxa, integrating network data with genomic sequences allows one to make some inferences about the evolution of the network itself. Thus, early work showed that gene duplication had a vital role in network evolution [2], with the redundancy created by that duplication decaying quickly [3]. These results led to the natural question of the influence of protein interactions on the patterns of gene duplication. It now appears that proteins residing in the less dense parts of the protein interaction network are more likely to duplicate [4]. In yeast in particular these duplication effects also depend on the type of duplication, with duplicated genes resulting from genome duplication tending to have more protein interaction partners than those produced by duplications of one or a few genes [5], [6].

It is also possible to assess how interaction networks change in time through genome comparisons. These approaches use the presence of an interacting pair of genes in outgroup genomes to identify the most ancient point at which a particular protein-protein interaction (or PPI) could have originated. The range of comparisons of this type are quite varied, from structural approaches that encompass the three domains of life [7], [8] to within-eukaryote [9] and within the fungal-animal clade comparisons [10]. In particular, Beltrao and Serrano [10] were able to use these phylogenetic signals in combination with the rate of divergence between duplicated genes in protein interaction networks to estimate a rate of link change in the protein interaction network of approximately 10^−5^ interaction changes per protein per million years. Interestingly, this number is reasonably similar to what was found in a more recent analysis that focused on experimental determination of selected interactions in several species of yeast, e.g., roughly 10^−4^ changes per million years [11].

Another vein of network research is assessing how protein interactions influence sequence evolution, especially how they alter selective constraint (i.e., the degree to which certain interaction-disrupting polymorphisms are filtered out of a population by purifying selection). Considerable work has gone into identifying predictors of these constraints (which, given a fixed outgroup, are sometimes described in terms of rates of evolution). Fraser and colleagues [12], [13] found that proteins with more protein interactions tended to evolve more slowly than those with fewer interactions. The hypothesized mechanism for this constraint is that proteins with many interaction partners have a larger proportion of their amino acid sequence in conserved binding sites. However, even this association is disputed: at best, it is rather weak [14], [15], [16]. In keeping with this observation, it is also known that residues on a proteins surface show lower selective constraint than do internal ones, probably because the latter contribute more directly to proper protein-folding [17], [18], [19], [20], [21]. In mammals it appears that many surface residues evolve essentially neutrally. Yet these proteins do not have fewer protein interactions than do proteins with more constrained surfaces [22]. This result, however, must also be qualified, since there is also evidence that surface residues involved in permanent protein interactions evolve slowly, while those involved in transient protein interactions have increased substitution rates [17]. In mammals [23] and in yeasts [24], it has also been found that extracellular proteins evolve faster than intracellular proteins. However, in yeast, it is difficult to tease apart the importance of cellular localization from gene essentiality [24]. In the end, though, all of these observations are somewhat immaterial, as gene expression still appears to be by far the strongest predictor of selective constraint [25], [26], [27], [28], [29]. Drummond and Wilke argue that this slow evolutionary rate of highly expressed proteins is due to the fitness costs of protein misfolding being greater for highly expressed genes [30].

One possible reason for a general lack of association of interaction and constraint is that protein interactions are instead inducing co-evolution between the two molecules [12]. In fact, under some models, such co-evolution may be sufficient to result in misleading phylogenetic signals due to correlated substitutions between interacting proteins [31]. Here again, however, the fact that interacting proteins also tend to be co-expressed may drive the similarities in rates of evolution [32]. Thus, the degree to which interacting proteins undergo co-evolution between their binding sites (i.e., compensatory substitutions that maintain the PPI) is still debated. Hakes and colleagues [33] argue that since interacting proteins experience the same environment and gene expression levels, the correlation in their evolutionary rates is sufficiently explained by these factors without needing to invoke correlated substitutions. However, known examples of co-evolution include the reciprocal changes between interacting residues found in the V3 loop of the human immunodeficiency virus (HIV) type 1 envelope protein gp120 [34], [35] and between the V3 loop and co-receptor binding domain of gp120 and the host cell’s CD4 receptor [36]. From a practical standpoint, using paired phylogenetic trees and shared changes to computationally identify PPIs is agnostic as to the reason for those paired changes and has been used to predict PPIs in both prokaryotes [37], [38] and eukaryotes [33].

**Figure 1:**
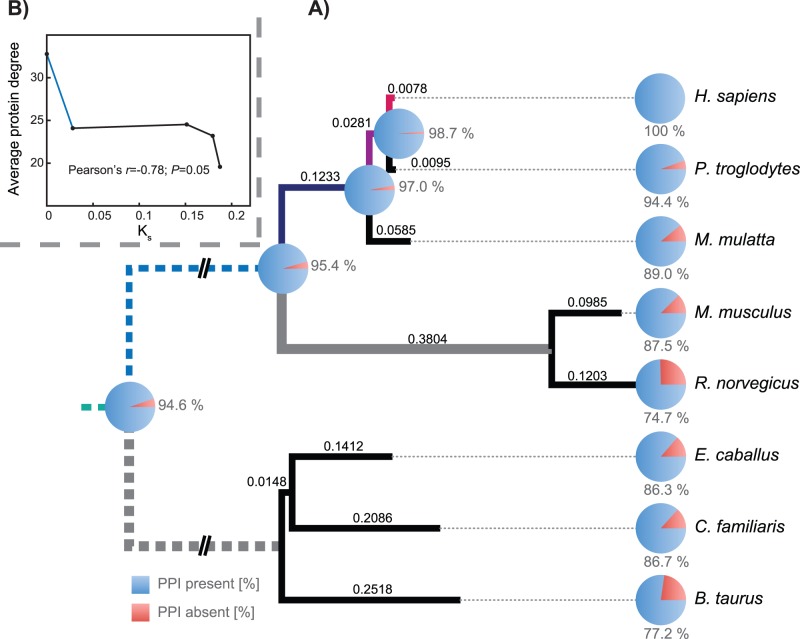
PPI presence and absence at the different nodes in the rooted eutherian phylogenetic tree. A) At each node, we have shown the predicted percentage of human PPIs present at that node (necessarily 100% at the human tip). The percentages at the other seven tip nodes were inferred by the presence or absence of the orthologs of the two human proteins making up the PPI (*Methods*). We then inferred the states of the internal nodes under the assumption that a given PPI ortholog pair could appear only once in the phylogeny (*Methods*). The topology was visualized using FigTree [61]. Branch lengths are the mean K_s_ value (e.g., number of synonymous substitutions per synonymous site) found across the genes surveyed for that branch of the tree (See *Methods*). The five colored branches indicate potential origin points for a PPI under our limited parsimony model (*Methods*), while the two gray branches were used to estimate the rate of PPI *loss*. The dashed branches indicate the fact the K_s_ values could not be distinguished for these two branches because the models used produce unrooted trees. **B)** There is an association between the age of the branch along which a PPI appears (*x-*axis; estimated via K_s_ above) and the average interaction degree of the proteins that make up that interaction (*y*-axis). Note that the blue distance was estimated as one-half the K_s_ distance between the rodent-primate and horse-dog-cow clade in the unrooted topology of (**A**). See *Methods* for details.

Here, we are interested in two primary questions. First, to what extent are human protein interactions evolutionarily ancient? Second, what is the nature of the selection acting on the network structure of the human protein interaction network? To explore these questions, we used previously described human PPI data and inferred orthologous genes from seven other mammals (Figure 1). We reconstruct part of the history of this network, as well as looking for evidence of correlated evolution between interaction partners. In addition to finding strong conservation among the PPIs, we find signals of weak but statistically significant co-evolution among the interacting proteins as well as confirming previous work that showed a tendency of interacting proteins to be under similar selective constraint [29].

## Results

### Inferring the Origins of the Human Protein Interaction Network

Given a set of previously described human protein-protein interactions, or PPIs [39], we identified the orthologs of the genes involved in each PPI from seven other mammals (*Methods*). We then inferred the earliest potential origin of each PPI (i.e., the earliest point at which orthologs of both genes involved were inferred to be present; *Methods*) using the phylogeny in Figure 1 and assuming that an orthologous gene could only appear once on that tree. Because our analysis started with a set of human PPIs, all such PPIs are present along the human branch in Figure 1. Strikingly, however, even at the base of the tree, we infer that 95% of the gene *pairs* involved in the current interactions were present. Of course, the presence of orthologous genes is not direct evidence for the existence of a PPI, especially given that most human genes have orthologs in these seven species [22]. We thus performed five analyses aimed at assessing to what degree this ortholog conservation might also indicate PPI presence at the various nodes in Figure 1a.

First, we examined the differences in age between pairs of interactors. It is possible that these ages are biased in such a way that the PPIs must be more recent than the average age of the genes encoding them (in other words, if a substantial excess of PPIs involve the product of one ancient gene and one gene shared only by the primates, that would imply that many PPIs are actually more recent than the ages of their component genes would suggest). We thus compared the set of real PPIs to a set of random “pseudo-PPIs” consisting of gene pairs drawn from the same set of genes (*Methods*). There was no statistical difference between the inferred ages of the real pairs and the random ones (*P>*0.05). This result might seem trivial, but it demonstrates that the PPIs have maximal ages that are at least congruent with the set of genes they are drawn from.

Second, given this similarity in age, we could ask a more subtle question: does the existence of a PPI between a pair genes give rise to concerted patterns of gene presence or absence for those genes? To find out, we counted the number of instances where the two genes encoding a PPI pair were both present or both absent in a given species. Strikingly, among the real set of PPIs, there were significantly more of both cases than in the randomized datasets, which correspondingly had more cases where only one member of the pair was present (*P≤*0.01). The implication is that PPI pairings are (at least in some cases), real, ancient and selectively meaningful: if they were not, we would not expect to find an excess of cases where both are present or both are absent.

Third, we hypothesized that if ortholog ages were actually a useful proxy for interaction age, there should be a trend for older interacting ortholog pairs to involve proteins with higher numbers of total interactions. The intuition here is that if many of the interactions considered here are truly ancient, they will involve older proteins that have had a longer period of time to gain interactions, as is generally seen for interacting proteins [8]. Indeed, we found that the average interaction degree of a protein is inversely correlated with the age of the branch where it appears (Figure 1b). Random networks (created as above) do not generally show this correlation (*Methods; P = *0.019).

Fourth, we used a sequence evolution-based correction to our estimates of PPI origin points. It is obvious that new PPI could easily evolve between two existing orthologs, but it is difficult to assess the magnitude of this error, given that existing PPI networks in other species are likely sparsely sampled as well. Instead, we used a *steady state* approximation to see if the rate of inferred PPI loss (through loss of orthologs) differed greatly from the inferred rate of gain (Figure 1a; Methods). After correcting for the length of each branch in Figure 1a using synonymous divergence, we found that the rate of PPI loss per PPI per unit K_s_ was 0.133 on the shared mouse/rat branch and the rate of gain per PPI per unit K_s_ (on the shared primate branch) was also 0.133. This similarity in value is obviously coincidental, but even if the *lowest* gain rate on the tree (shared primate branch; 0.133) is compared to the highest loss rate (0.84 on the shared horse/dog branch), our estimate of the number of human PPIs present at the root of Figure 1a only drops to 74%. Thus, it appears that our estimate of PPI ages may be relatively robust to the use of orthology data.

Finally, we examined patterns of co-evolution to see if they supported an ancient origin for most human PPIs. If a PPI is selectively important, one might expect there to be correlated substitutions between the interacting proteins in order to maintain that interaction. For our set of PPIs, we thus calculated the correlation coefficient between the paired, branch-specific, selective constraints for each pair of genes corresponding to a PPI. We compared the mean Spearman’s ρ from this set of true PPIs (t-PPI) set against the distribution of means seen for 1000 random datasets of the same size (pseudo-random or pr-PPIs; *Methods*). None of the mean Spearman’s ρs from the pr-PPI datasets were as large as that observed in t-PPI (Table 1). Omitting branches with ω≥5.0 from the analysis did not alter our conclusions. Thus, there is evidence for correlated evolution among the interacting proteins. We therefore sought to use this correlation to assess whether the PPIs considered were ancient. We asked whether the correlation in selective constraint between interacting genes tended to be weaker in parts of the tree most evolutionarily distant from humans, as one would predict if there were a significant number of recently evolved PPIs in these data. We thus sequentially removed several clades from the phylogeny of Figure 1a and repeated our co-evolution analysis. In general, our results were in accord with expectations: the difference between the mean correlation from t-PPI and the mean of means in the pr-PPI datasets increased when more distant taxa were omitted from the analysis (Table 1). Nonetheless, these branches are contributing to the co-evolution signal, as omitting several of them together reduces that signal considerably (Table 1). Thus, there is evidence that co-evolution indicative of conserved PPIs is present even at deep nodes of the phylogeny.

**Table 1:** Coevolution between PPI partners detected using correlated changes in selective constraint.

Dataset/ω cutoff^a^	Clade removed^b^	*#PPIs* c	*P* d	Mean Spearman’s correlation (Real data)	Mean of means (Spearman’scorrelation,1000simulations)^e^	Difference
Full data set: 0≤ω<∞	None	7730	*<0.001*	0.131	0.122	0.009
0≤ω<5	None	7727	<0.001	0.132	0.122	0.009
0≤ω<5	Human	7705	<0.001	0.123	0.110	0.013
0≤ω<5	Chimpanzee	7668	<0.001	0.102	0.099	0.003
0≤ω<5	Macaque	7303	<0.001	0.126	0.108	0.018
0≤ω<5	Mouse	7173	0.007	0.132	0.128	0.004
0≤ω<5	Rat	7132	0.003	0.137	0.132	0.005
0≤ω<5	Horse	6937	0.001	0.139	0.131	0.008
0≤ω<5	Dog	6930	0.005	0.135	0.128	0.007
0≤ω<5	Cow	6785	0.011	0.133	0.127	0.006
0≤ω<5	Human/Chimp	7563	<0.001	0.095	0.084	0.011
0≤ω<5	Primates	6113	<0.001	0.070	0.054	0.016
0≤ω<5	Rodents	5123	0.091	0.106	0.111	−0.005
0≤ω<5	Horse/Dog	5893	0.061	0.141	0.138	0.003
0≤ω<5	Horse/Dog/Cow	3456	0.421	0.165	0.169	−0.004

a Values of branch-wise selective constraint (ω) allowed in the computation of Spearman’s correlation between these ω values between paired branches for two proteins with a known PPI in humans (*Methods*).

b Values of ω from the indicated clades were removed before the calculation of the Spearman’s correlation.

c We required at least 6 common branches between the two orthologous genes trees for the two interacting proteins: the column indicates the number of PPIs meeting this requirement.

d P-value of the hypothesis test that the real PPI pairs had a higher mean Spearman’s correlation than would be expected, given the distribution of correlations seen from 1000 simulations of the same number of pseudo-PPI pairs drawn from non-interacting proteins (*Methods*).

e Mean of the mean correlation seen from 1000 simulations, each consisting of the same number of pseudo-PPIs from *c*.

### Functional Annotation of Primate-specific PPI Genes

**Figure 2:**
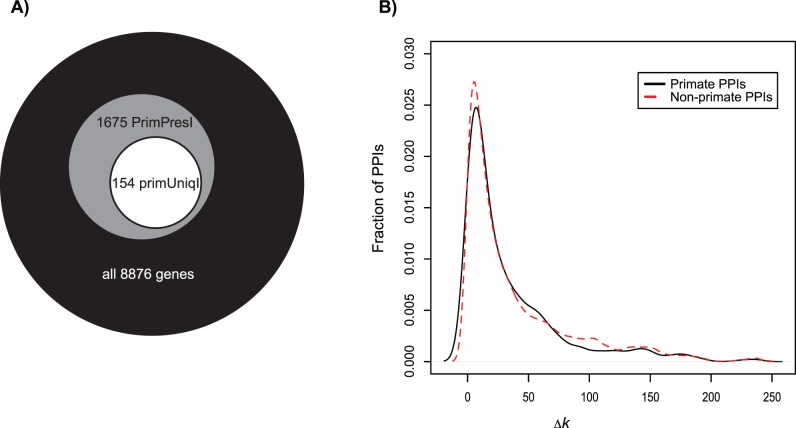
Differences between primate-specific and phylogenetically-distributed interactions. A) Gene sets used in the GO analyses of primate-specific protein interactions. There are 8876 human genes having at least one interaction (for a total of 32,916 PPIs). Among those genes, 1502 interactions (encoded by 1675 genes) are found only in primates. Of those 1675 genes, 1,521 are also involved in other, nonprimate-specific interactions, and 154 are only involved in primate specific interactions. **B)** Genes involved in primate-specific interactions have, on average, more total interactions (i.e., the genes involved in these interactions tend to have a higher degree *k*). The distribution of the difference in degree (*k*) for each gene in a pair of interaction proteins was compared (here referred to as ‘absolute degree difference’, Δ*k*; *x*-axis). In black are the primate-specific interactions (primatePPIs) while red (dashed-line) shows the remainder of the interactions.

We next sought to explore the functional roles of some of the putatively recently evolved PPIs. Thus, we performed GO analyses to explore the role of the primate-specific PPIs (*Methods*). We first compared 1675 genes that were present in at least one primate-specific PPI (and potentially also in nonprimate PPIs; e.g., PrimPresI) to the 7201 genes that were not involved in a primate-specific PPIs (Table 2, Figure 2a). We found that the genes from PrimPresI were over-represented for biological process GO terms including “cell death,” “cell communication”, “response to stimulus,” and “macromolecule metabolic processes”, while no biological process GO terms were under-represented. Over-represented molecular functions included “protein binding,” “signal transduction activity,” “transferase activity,” and “kinase activity” while “oxidoreductase activity” was under-represented (Table 2). Using the same PPIs we also compared 154 genes involve *only* in primate-specific PPIs (i.e., these genes are not part of any nonprimate PPI; PrimUniqI) against the remaining 8722 genes. No GO terms were over- or under-represented in this dataset.

**Table 2:** Over- and under-represented GO terms of genes present at least once in a primate-specific PPI.

Class	ID	GO term	#Obs^a^	#Exp^b^	*P* c	Fold excess
Biological process	0006139	nucleobase-containing compound metabolic process	518	467.7	4.0×10^−2^	1.21
Biological process	0007154	cell communication	692	528.8	9.6×10^−21^	1.51
Biological process	0007275	multicellular organismal development	403	353.5	1.6×10^−2^	1.26
Biological process	0008219	cell death	230	149.4	4.1×10^−12^	1.89
Biological process	0009987	cellular process	989	916.3	3.5×10^−4^	1.17
Biological process	0030154	cell differentiation	256	193.7	4.4×10^−6^	1.53
Biological process	0032501	multicellular organismal process	246	207.9	3.3×10^−2^	1.32
Biological process	0043170	macromolecule metabolic process	893	781.6	4.2×10^−9^	1.26
Biological process	0050789	regulation of biological process	1011	865.8	8.4×10^−16^	1.30
Biological process	0050896	response to stimulus	385	314.3	1.4×10^−5^	1.39
Biological process	0051704	multi-organism process	119	79.6	2.7×10^−5^	1.81
Molecular function	0004871	signal transduction activity	137	74.1	4.9×10^−13^	2.39
Molecular function	0005515	protein binding	1264	1065.8	1.6×10^−33^	1.28
Molecular function	0016301	kinase activity	183	116.3	3.0×10^−10^	1.90
Molecular function	0016491	oxidoreductase activity^d^	27	54.6	1.3×10^−4^	0.45
Molecular function	0016740	transferase activity	199	163.9	3.9×10^−2^	1.33

a Observed instances of the GO term. 1675 genes present in primate PPIs vs 7201 genes never observed in primate PPIs.

b Expected number of occurrences among an randomly-selected set of genes of the same size.

c *P*-values for the test of the hypothesis of no difference between the observed and expected number of occurrences of the term after a Bonferonni multiple-test correction.

d Term was *under-represented* among the primate-specific PPIs.

### Protein Degree of Primate-specific PPI Genes

We also asked if the proteins involved in (recently-evolved) primate-specific interactions differed in their degree (number of interactions, *k*) from the remainder of the network. Proteins participating in a primate-specific PPI (i.e., the PrimPresI dataset; *Methods*) have a significantly higher mean interaction degree than other proteins (Table 3). The obvious interpretation is that genes of high degree are also more likely to have a primate-specific interaction by chance. To explore this possibility, we compared the connectivity of genes with only primate-specific PPIs (PrimUniqI) to all other genes. Again, there is a significant difference: but in this case, the PrimUniqI genes had *fewer* interactions (Table 3), again because the restricted set of genes with only primate specific interactions would tend to have low degree. We also examined the most highly connected protein in each set: Amongst the primate genes, the YWHAG gene (ENSG00000170027) has the product with the highest degree (*k = *240 including one self-interaction) and encodes a tyrosine 3-monooxygenase/tryptophan 5-monooxygenase activation protein [28]. The product of Ensembl gene ENSG00000170312 has the highest degree amongst the nonprimate set with k = 110 (no self-interaction). It is annotated as a CDK1 cyclin-dependent kinase 1. Finally, amongst the genes that exclusively participate in primate-specific PPIs, the protein of highest degree is encoded by ENSG00000198400 (a TRK1 neurotrophic tyrosine kinase receptor type 1; *k* = 31, including one self-interaction).

**Table 3:** Connectivity statistics of genes involved in primate PPIs vs genes part of nonprimate PPIs.

Measure	Primate genes	Nonprimate genes	Primate-specific genes^a^	All other genes^b^
k_min_	1	1	1	1
k_max_	240	110	31	240
k_mean_	18.6	5.1	1.8	7.7
*P* c	2×10^−16^		2×10^−16^	

a Set of genes involved *only* in primate-specific interactions.

b All genes not in (*a*).

c Wilcoxon test.

Proteins in general tend to interact with proteins of a different degree. We therefore investigated if this trend was consistent between the primate-specific PPIs (‘primPPIs’), and the nonprimate PPIs (‘nonprimPPIs’). For this purpose we calculated the absolute degree difference for the two constituent proteins across all interactions in the two sets:
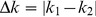
(1)where *k*_1_ and *k*_2_ are the degrees of the proteins in question. The average degree difference for primate-specific PPIs, Δk_primPPIs_, is 34.8, as compared to 38.9 for nonprimate PPIs, a significant difference (*P* = 6×10^−4^, Wilcoxon two-sample test; Figure 2b). Note however, that the maximum difference in degree for the two datasets was the same (239) due to the presence of the highly interacting protein YWHAG in both PPI datasets.

### Weak Evidence for Shared Instances of Adaptive Evolution between PPI Partners

**Figure 3:**
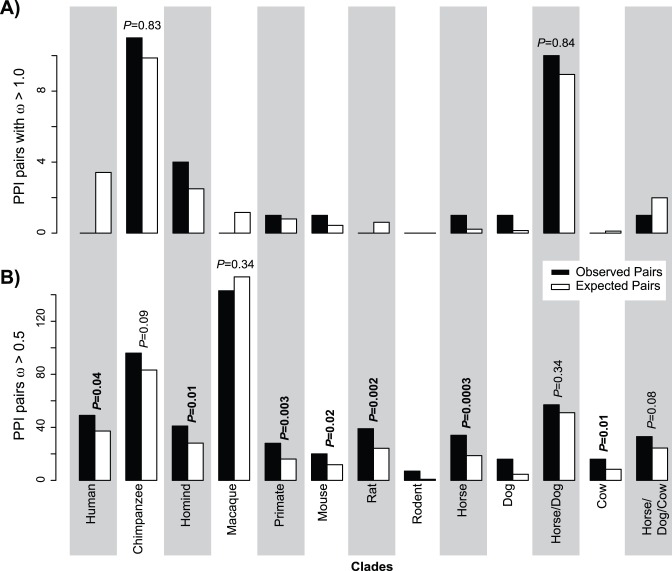
Paired cases of relaxed selective constraints for PPI pairs. For each clade in Figure 1, we plot the number of cases where both members have either ρ>1.0 (**A**) or >0.5 (**B**). *P*-values are shown for the test of the hypothesis that there are more such shared cases of relaxed constraint than would be expected by chance (χ^2^ test, *Methods*). Cases where no *P*-value is shown had too few observations of ρ>5 for valid statistical conclusions to be drawn.

We sought to assess if there were pairs of genes involved in a PPI that both shared an instance of adaptive evolution (e.g., ω>1.0) along the same branch of the phylogeny. However, the number of such paired cases of ω>1.0 was not significantly greater than what would be expected given the overall number of cases where ω>1.0 (Figure 3a). However, when we lowered the threshold to ω>0.5, we found that, with exception for macaque, all branches we observed more such cases than we would expect by chance (Figure 3b). We therefore performed a GO analysis comparing the 524 genes that had paired branches in the mirrortrees with ω>0.5 to the remaining set of genes without such signals. These 524 genes were over-represented for biological process GO terms such as “cell death” and “response to stimulus”, and the molecular function terms “protein binding” and “receptor activity” (Table 4; ‘AdaptI’ dataset, see *Methods*); no under-represented GO terms were found.

**Table 4:** Over- and under-represented GO terms of genes present in PPIs where proteins in the protein pair have ω>0.5 for both branches vs remaining 4506 genes.

Class	ID	GO term	#Obs^a^	#Exp^b^	*P* c	Fold excess
Biological process	0008219	cell death	75	45.6	9.9×10^−5^	1.81
Biological process	0050789	regulation of biological process	289	257.1	3.0×10^−2^	1.15
Biological process	0050896	response to stimulus	142	86.3	5.3×10^−10^	1.81
Biological process	0051704	multi-organism process	39	21.4	3.4×10^−3^	2.03
Molecular function	0004872	receptor activity	71	49.4	2.7×10^−2^	1.55
Molecular function	0005515	protein binding	384	336.5	8.0×10^−6^	1.18

a Observed instances of the GO term. 524 genes with ω>0.5 for both branches vs remaining 4506 genes (of 5030 genes in total from 12472 PPIs for which mirrortrees could be constructed with reliable ML scores).

b Expected number of occurrences among an randomly-selected set of genes of the same size.

c Uncorrected *P*-value for the test of the hypothesis of no difference between the observed and expected number of occurrences of the term.

### Proteins Interact with Other Proteins of Similar Constraint more Often than Expected

To further explore the type of shared similarity in selective constraint between PPI partners seen above, we developed a network-based test of whether selective constraints are distributed at random in the PPI network (see *Methods*). Briefly, we compared the selective constraint, ω, between pairs of interacting proteins. The distribution of selective constraint in the network is highly non-random: the average difference in selective constraint between two interacting proteins is 0.10, as compared to an average of 0.12 seen when the ω values are distributed at random (*P<*0.0001; *Methods),* confirming the results of Vinogradov [29], obtained with a different approach.

## Discussion

Our analysis suggests the slightly unexpected conclusion that most human protein-protein interactions are actually evolutionarily ancient (i.e., shared with most placental mammals). However, this conclusion is in accord with the work of Qian and colleagues, who estimated based on preservation rates of PPIs in yeast that a human protein interaction has a 98% chance of also being present in mouse [11]. Although their estimation of PPI presence of 98% is substantially higher than the 87.5% estimated here from our parsimony analysis, the two analyses are in general agreement that most PPIs are ancient. Given this conclusion, it is not surprising that Pellegrini and colleagues were able to use genes’ phylogenetic profiles predict protein interactions in yeast species [40], [41].

Of course, our approach is nevertheless potentially biased by the fact that we assess orthology and not interaction directly. Thus, we are in fact setting an upper bound on the number of interactions present at older nodes in the tree. One might wonder why one would bother to use orthology data to study this type of network at all. The basic reason is that all known protein interaction networks appear to be very sparely sampled [42], [43]. Thus, estimating interaction evolution rates even between human and mouse (the only other mammal with extensive PPI sampling) will be very difficult. Worse, the PPIs known from human and mouse are not independent, potentially introducing a bias. In the future, it may be possible to computationally predict the possibility of an interaction across multiple genomes [43], but even such an approach needs to be validated with evidence for the actions of selection to maintain those interactions. Likewise, the work of Qian and coauthors very elegantly estimates interaction evolution rates, but does not evaluate the network as a whole [11]. Given these issues, there remains a niche for orthology-based analyses of interaction. Similarly, one might think that some of the interactions included might be due to various types of false positives for interaction presence. However, because our approach is based on collective statistics regarding the interactions, it is unlikely that elimination of those false positives from the interaction dataset would alter our results. We also note that the HPRD data used appears to show a good balance of comprehensiveness and quality [44].

Despite the biases in our parsimony analysis, our overall conclusion that most human protein interactions are ancient is supported by a second analysis, which shows that there is a signal of co-evolution among PPI pairs, even among mammals that are reasonably distant evolutionarily from humans (e.g., horse, dog and cow). Our analysis involved comparisons of the selective constraint along matched branch pairs for interacting proteins (Figure 1a and Figure S1). Co-evolution has been previously used to identify sites with interacting residues but many of the methods used are site-specific and require three-dimensional protein structures [36], [45], [46]. Other methods detect co-evolution by calculating the pairwise distances between sequences of proteins known to interact, the values of which are then used as references for predicting other interactions [37]. Interestingly, although the ‘binding neighborhoods’ of interacting proteins give the strongest co-evolutionary signal, co-evolution can also be detected at other sites [47]. Co-evolution can also extend beyond direct protein interactions: only after the network distance between two proteins exceeds 3 is the co-evolution signal lost [48].

This co-evolution analysis also sheds light on our second question, that of the selective forces acting on protein interactions. These data suggest significant and abiding selection that acts to maintain interactions. This conclusion is supported by our finding that the difference in average selective constraint between interacting proteins is smaller than expected, a result consistent with the findings of Vinogradov [29], made with a completely distinct approach. The obvious question raised by these observations is the exact nature of the selection at work. Fernandez and Lynch have recently argued that many of the protein interactions in multicellular eukaryotes originated not through selection for novel functions but rather as a means for stabilizing existing functions in the face of the destabilizing forces of genetic drift [49]. No results in the present work contradict this hypothesis as a general principle, but there are two points that suggest that it is probably not an exclusive process. First, co-evolution is not a strong prediction of a model where specific interactions are not under selection but instead there is generalized selection for enough interactions to maintain protein function. Second, we have (very weak) evidence for shared instances of directional selection in interacting pairs. The variety of conclusions and models for understanding protein interactions appears to suggest that our understanding of these processes is still immature and that new, *predictive,* models of these networks are needed.

Our analyses of the phylogenetic and selective patterns observed among mammalian protein-protein interactions supports a model of interaction conservation through some degree of purifying selection. There are of course wide error bounds on these estimates, making it inappropriate to depend on them for a given interaction. But in general it appears that the evolution of the protein interaction network may not be as rapid as earlier believed.

## Methods

### Estimating the Time of Origin of the Human Protein Interactions

We employed a set of human PPIs described previously [39], consisting of 32,916 interactions among 8876 genes. Self-interactions were excluded. We identified the orthologs of these 8876 genes from seven other mammals (*Pan troglodytes, Macaca mulatta, Mus musculus, Rattus norvegicus, Equus caballus, Canis familiaris and Bos taurus*, Figure 1a) using a previously described approach [22]. Thus, we first identify homologous genes using our GenomeHistory program [50]. One-to-one relationships among the homologs of a pair of genomes are assumed to be orthologs. Further orthologs are identified by breaking multigene families, assuming that homologous neighbors of existing orthologs are also orthologs (e.g., a synteny-based approach).

From these data, we inferred whether each PPI could potentially exist in the seven other species, given their ortholog complements (if either ortholog is missing, so necessarily is the interaction). We coded the status of each PPI in each species as:

- 4 if both orthologs were present
- 2 if the ortholog for the first (human) gene was absent
- 1 if the second ortholog was absent.
- 0 if both were absent.

These data are unusually structured in several respects. First, by definition, all interactions are present in humans. Second, because our orthology identification rests on both sequence and gene order data, independent appearances of the same state are vanishingly unlikely. Third, we are limited to detecting the presence of the orthologs of the two interacting genes in the other seven species: we have no direct way of assessing if the interaction itself is present. Finally, we may identify an ortholog as missing either due to true evolutionary loss or due to issues with annotation or orthology-calling. For all of these reasons, standard parsimony approaches are inappropriate. Instead, we sought to identify the *latest point* on the phylogeny in Figure 1a at which a given interacting pair of genes could have appeared, given the orthology data. This problem devolves into placing the origin of the interacting pair on one of the five colored branches in Figure 1a, all of which are along the lineage leading to human (because we started with known human interactions). Using this approach, we estimated the number of appearances of interacting orthologs along these branches (circles in Figure 1a).

### Comparing the Age of the Protein Interactions to the Age of the Orthologs Involved (Validations #1 and 2 in Results)

As mentioned, dating the appearance of orthologs at best estimates the *maximum* age of a PPI: new interactions could easily evolve between ancient orthologs. We cannot directly compare interaction presence between different mammals due to a lack of data. However, we can at least indirectly assess if the age distribution of the *pairs* of orthologs that make up PPIs differs from the underlying distribution of ortholog ages. The logic here is that if PPIs are predominately of a recent origin, they should fall more often on later branches of the tree in Figure 1a than would an equivalent number of gene pairs sampled at random from the set of orthologs. To assess this possibility, we created sets of pseudo-PPIs drawn at random from the set of genes having PPIs: the probability of drawing such a gene was proportional to the number of interactions it had. No self-interactions or actual interactions from the PPI dataset were allowed in these datasets. We then repeated the inference of points of origin on the 100 random datasets. We also tested the similar hypothesis that whatever their age, pairs of genes involved in a PPI will be more likely to be either both present or both absent in a given taxa. To assess this possibility, we counted the number of instances of each of the four states above in the random datasets and compared those proportions to those from the real PPI data.

### Comparisons of the Average Degree Distributions of Proteins Involved in PPIs Appearing at Different Points in the Mammalian tree (Validation #3 in Results)

For each of the five nodes in the direct human lineage in Figure 1a (colored branches), we computed the average degree distribution for all proteins involved in interactions first appearing at that node. (Note that this calculation is *not* equivalent to calculating the degree of protein-coding genes appearing at this node: older genes may be included if they interact with a younger gene appearing at that node). We then compared the average degrees for these five nodes, finding that they generally increase as one descends to progressively more ancient nodes (Figure 1b, *Results*). Next, we created random PPI networks as discussed above and repeated this analysis, comparing the Pearson’s correlation from Figure 1b to that seen in the randomized networks. The real network had a higher correlation than expected (*P = *0.019).

### Comparison of Rates of Interaction Loss and Gain Based on Sequence Evolution (Validation #4 in Results)

To assess the degree to which using ortholog presence under-estimates the rate of PPI acquisition over evolutionary time, we compared the ratio of PPI gain to loss, calibrating the rate of the two types of event based on a sequence-based measure of time: the average number of synonymous substitutions per synonymous site (K_s_). These K_s_ values were estimated from the 5030 alignments analyzed with PAML 4.4 [51] as discussed below. We can best estimate loss rates from shared branches not containing direct ancestors of humans (thick grey branches in Figure 1a). If we assume that the protein-interaction network is in steady state (i.e., the number of edges is neither increasing nor decreasing in time), the loss rate per unit K_s_ should equal the gain rate per unit K_s_.

### Assessing Co-evolution between Interacting Proteins (Validation #5 in Results)

We next assessed the degree of co-evolution between pairs of interacting proteins by looking for shared changes in the selective constraint of their coding sequences. To do so, we used non-self PPIs with 1∶1 orthology across all 7 other species for both genes. Using sequence data from Ensembl release 50 [52], we aligned the orthologous proteins sequences with MUSCLE v3.6 [53], converted those alignments into nucleotide alignments and performed a number of alignment quality checks [54]. Alignments that passed these filters were analyzed with codeml (model M1) in PAML 4.4 [51], producing maximum likelihood estimates of the ratio of nonsynonymous to synonymous substitutions per site (e.g., K_a_/K_s_, hereafter referred to as ω) for each branch in Figure 1a. Such tree-based approaches produce better estimates of correlated evolution than pairwise sequence comparisons [55]. In order to avoid erroneous ω estimates resulting from codeml having become trapped in a local optima, we started PAML from random initial conditions until the three analyses with highest likelihood differed by no more than 5% in their estimates of ω. In three cases (ENSG00000110400, ENSG00000129038 and ENSG00000154767), the three top runs did not agree even after 100 iterations, and so these three genes were omitted. The result of this pipeline was estimates of ω for 5030 alignments, collectively involved in 12,472 PPIs.

### Estimating Co-evolution Using the Correlation of ω Values

The above estimates of ω allowed us to construct mirrortrees for each PPI, e.g., paired phylogenic trees, one from each protein [56]. These paired trees consisted of (a maximum of) thirteen paired estimates of ω per PPI (one per branch). To avoid the large sampling variances for cases where ω≥5, we also performed our analyses omitting branches with such high values of ω (Table 1). We computed the Spearman’s correlation coefficient, ρ, between those paired ω values, requiring a minimum of 6 paired branches in order to do so. The result was 7727 PPI pairs with associated correlations. As an aside, we note that because ω values have a highly non-normal distribution (a range from 0 to positive infinity but with a strong bias toward zero), we found that the Pearson’s correlation coefficient was subject to strong outlier effects (Figure S1).

To assess whether the correlations observed from the interacting genes were statistically significant, we compared the distribution of ρ from the true PPIs (t-PPI) to the distribution of ρ values seen in a set of similar, non-interacting, gene pairs created by randomization. To create these random datasets, we started by generating pairs of genes from the 5030 genes, requiring that the two genes in each pair were *not* an interacting pair. Sets of 7727 unique gene pairs with no true interactions among them and a minimum of six branches in common were defined as new datasets (pseudo-random interactions or pr-PPI). We compared the mean value of Spearman’s ρ from t-PPI to the distribution of 1000 means from the pr-PPI datasets. We also estimated the degree to which each species or clade contributed to the co-evolution signal by removing the orthologous genes from that species and repeating the above analysis (Table 1).

### Gene Ontology Analysis

Gene Ontology (GO) analysis was performed on three datasets (Figure 2a). The first two datasets were created from the PPIs used to infer the ancestral states of the PPI network (Figure 1a), while the third dataset was created from PPIs used for detecting signals of adaptive evolution:

1. Genes that participated in at least one primate-specific PPI, although not exclusively in primate-specific PPIs (hereafter primate-present interactions; PrimPresI)
2. Genes involved *exclusively* in primate-specific PPIs (hereafter primate-unique interactions; PrimUniqI)
3. Genes from PPIs for which mirrortrees had both branches in any given species with molecular rates ω>0.5 (hereafter adaptive interactions; AdaptI)

All three datasets genes were matched to GO slim [57] via conversion of Ensembl IDs to human GO identifiers [58], which were obtained from the Gene Ontology website (http://www.geneontology.org) [59]. *P*-values for GO terms were calculated under the hypergeometric distribution and adjusted for multiple tests with a Bonferroni correction. We also asked if the degree distribution *k* (the number of interactions) differed between the above three gene sets and the rest of the network.

### Shared Signals of Adaptive and Co-evolution

We hypothesized that there might be cases of shared adaptive evolution among the PPI pairs. To explore this possibility, we looked for shared cases of ω>1.0 in paired branches from the mirrortrees using a χ^2^ test. A similar analysis was performed with a threshold of ω>0.5, under the assumption that directional and purifying selection might have occurred on the same branch, limiting the divergence signal.

### Association of the Degree of Selective Constraint and Protein Interaction Network Position

We have previously analyzed the selective constraints of 13,928 sets of mammalian orthologs [54]. Briefly, these data consist of estimates of per-alignment estimates of ω, calculated with codeml (model M0) in PAML 4.2 [51] (i.e., not *per* branch as above). Each such alignment includes one human gene and a minimum of six other mammalian orthologs [22], no of which were allowed to have any tandem duplications [54]. We used these data to ask if proteins of similar selective constraint were more likely to interact with each other. We first reduced the set of human PPIs to only those interactions where both proteins were found in the above orthology set. We then created a PPI network where each protein node was weighted by its value of ω: denoted n_ω_. Consider two interacting nodes *n* and *m*. We define the edge weight, e_Δω_, for that interaction as:
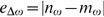
(2)


We then calculate the average edge weight, *w*, of the entire network with:
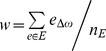
(3)where *E* is the edge set of the network and *n_E_* is the number of edges.

### Statistical Analysis of the Network Weights

To assess if the average weight was smaller than would be expected by chance, we randomly reassigned the set of node weights *n*_ω_ 10,000 times and recalculated the value of the weight (*w_R_*) for each of those random networks. We then asked where *w* from the real network fell in the distribution of *w_R_*.

One might think that the signal of similarity in selective constraint is an artifact of a few protein complexes that define many pairwise PPIs as well as having a similar selective constraint across the complex. Were this situation the case, we would expect that interacting nodes would be similar not only in their constraint but also in their connection degree. To test this possibility, we applied a modification of the above approach, making the edge weight for a pair of nodes the difference in their respective degrees (number of PPIs). We then calculated the overall network weight as before. We compared this weight to the weight seen for a distribution of 1,000 randomly rewired PPI networks that have identical node degrees but where the interaction identities have been scrambled [60]. Contrary to the above prediction, proteins in the real network are *less* likely to interact with nodes of a similar degree (*P = *0.013).

## Supporting Information

Figure S1 **Distributions of Pearson’s and Spearman’s Rank Sums correlation coefficients of the true PPIs and pseudo random PPIs respectively.** The true (solid lines) and pseudo random (dashed lines) PPIs. A) Distribution of the Pearson’s correlation coefficient *r* includes a ‘bump’ at approximately *r* = 0.7, indicating that the data are non-normally distributed. This is caused by branches that are outliers compared to other branches in some of the mirrortrees, which then inflates Pearson’s *r*. That these correlations are spurious is suggested by the fact that no similar bump is seen in B), the Spearman’s rank sums correlation coefficients ρ for the same data.
(EPS)
